# Supplementary material for: Exploring 3D miniatures with action simulations by finger gestures: Study of a new embodied design for blind and sighted children
Source: PLoS One. 2021 Feb 3;16(2):e0245472. doi: 10.1371/journal.pone.0245472 (PMC7857736; doi:10.1371/journal.pone.0245472)
Supplement: S1 Table — (DOCX) [file pone.0245472.s001.docx]

S1 Appendix

| **Mean scores of contact frequency (max 4.00) and (SD) with action objects tested in sighted (N=15) and blind children (N=8) - Experiment 1. Objects are listed by order of frequency for each group of subjects.** | | | | | | |
| --- | --- | --- | --- | --- | --- | --- |
| Action Objects (listed by order of frequency) | Sighted | |  | Action Objects (listed by order of frequency) | Blind | |
| Stairs | 3.93 | (0.26) |  | Stairs | 3.75 | (0.71) |
| Toboggan | 3.13 | (1.13) |  | Toboggan | 2.38 | (1.69) |
| Swing | 2.53 | (1.30) |  | Swing | 2.13 | (0.64) |
| Bicycle | 2.07 | (1.16) |  | Bicycle | 1.50 | (1.20) |
| Merry-go-round | 1.47 | (1.19) |  | Trampoline | 1.13 | (1.25) |
| Trampoline | 1.07 | (0.80) |  | Merry-go-round | 0.88 | (0.83) |
| Roller Skate | 1.07 | (1.16) |  | Roller Skate | 0.25 | (0.46) |
| Total | 2.18 | (0.44) |  | Total | 1.71 | (0.64) |
