# Supplementary material for: Exploring 3D miniatures with action simulations by finger gestures: Study of a new embodied design for blind and sighted children
Source: PLoS One. 2021 Feb 3;16(2):e0245472. doi: 10.1371/journal.pone.0245472 (PMC7857736; doi:10.1371/journal.pone.0245472)
Supplement: Attachment — Submitted filename: Response to reviewers.pdf [file pone.0245472.s004.pdf]

Response to reviewers for "Exploring 3D miniatures with action simulations by finger gestures: study of a new embodied design for blind and sighted children" (PONE-D-20-13459)

We would like to thank the editor and the reviewers for their constructive comments. We addressed all the points raised by the reviewer and editor and explained how below. In the manuscript, all changes are marked in blue.

Journal Requirements:

1. Please ensure that your manuscript meets PLOS ONE's style requirements, including those for file naming. The PLOS ONE style templates can be found at

https://journals.plos.org/plosone/s/file?id=wjVg/PLOSOne_formatting_sample_main_body.pdf and

https://journals.plos.org/plosone/s/file?id=ba62/PLOSOne_formatting_sample_title_authors_affiliations.pdf

Answer :

In title page, country of authors are included. We proceeded to a verification of others items and style requirements are respected.

2. Please state in your methods section whether you obtained consent from parents or guardians of the minors (those aged <18) included in the study or whether the research ethics committee or IRB approved the lack of parent or guardian consent.

Answer :

The statement "Parents or guardians provided signed written informed consent forms for their children to participate in the experiment " was included for Experiment 1 (page 10, line 205) and for Experiment 2 (page 18, line 369)

Reviewer #1:

Reviewer #1 : There is one overarching question related to the motivation presented by the authors that relates to the number of actions that can be presented with ASFG. It would be relevant to understand how this procedure can be applied beyond the limited set of actions presented. I am sure there are others, but question if they are representative in the large number of objects available in children's books. This is not a deal breaker for the specific contribution that is offered in this manuscript, but something that this reviewer finds relevant to see

discussed given the context that is offered in the introduction.

Answer:

Thank you for raising this very interesting point. Indeed, although we explain in the Method section (page 10, line 211) the criteria on which we based our selection of the 7 ASFGs in this study, it is very important to show how these conclusions drawn from a limited set of actions can be extended to other illustrations included in books for blind children. We therefore further develop this point in General Discussion:

Page 24, line 543: The central question of our study is the contribution of embodied cognition and sensorimotor experiences in blind and sighted childrens' identification of illustrations. The advantage of 3D scenarios that engage ASFGs over traditional 3D miniatures (toys or miniature objects already commonly used in this field of intervention) is the reactivation of sensorimotor components by a gesture simulation (two fingers that act as two legs). As mentioned, this procedure was validated during the first phase of research in which a set of 18 ASFGs have been studied [25]. In the present study, we tested only 7 of these 18 following a feasibility criterion due to the design and nature of the study. Specifically, the ASFGs included should be reproducible in 3D miniature and also represented as 2D content. However, it is important to note that our previous study [25] showed that several other ASFGs are also very prototypical (same gestural pattern in the congenitally blind, late blind and sighted adults) such as "Kicking a ball", "Jumping on one leg" or "Walking backwards". These other ASFGs can be included in a book for blind children even if they have not been tested here.

Let us also add that the contribution of the embodied cognition that we have awakened via the ASFG procedure is not limited to the use of the ASFG. An example from our own study is the illustration of the toboggan as a textured picture that seems to be better recognized than the other illustrations due to the possible role of the sensorimotor experience reactivated when hands explore the slope of the toboggan. Other examples of illustrations involving sensorimotor or perceptual experiences other than visual ones can be found in books for blind children. For example, in the book entitled Wa-Wa (2012) about toilets published by the non-profit publishing house Les Doigts Qui Rêvent, children can flush the toilet thanks to an interactive system and a cord attached to the page. In A Magic Winter (2012) also published by Les Doigts Qui Rêvent, the child can walk on the snow, which is "illustrated" by a texture reproducing the sound and the sensation under the feet. The book "We're going on a bear hunt" (2015) designed with our scientific support [48] has a sliding mechanism allowing the reader to move a character to the other end of the page. In the very interactive book "The three little pigs" (2019) published by the non-profit publishing house Mes Mains en Or, the reader blows down pop-up houses with his breath.

Following direct feedback from child readers and their entourage who seem to appreciate this content more than the visual content in relief, some designers of tactile books are already choosing illustrations that allow for manipulation of objects, sounds and reactivation of haptic and sensorimotor experiences. The major contribution of our study is to provide scientific evidence of the role of these experiences in the identification of

objects by touch, regardless of the visual status of the children. A feasibility study is already underway with our editorial partnership (non-profit publishing house Les Doigts Qui Rêvent) to evaluate the possibilities of including ASFGs in tactile devices such as children's books but also in mobility aids.

Reviewer #1: The major concern I have with the paper is the low sample size in each of the experiments, particularly considering visually impaired children (n=8 in each experiment). Adding to that, the experiments were also performed with different children. Although the paper does not try to explicitly and statistically compare results between the two "experiments", it implies that recognition are better with ASFG than with Textures. Given the variability of children with visual impairments, and that not attempt to match the experimental and control groups was made, it is impossible to say something about this comparison. The results within each experiment are interesting, but given the limited sample sizes, they need to be taken into account carefully.

Answer:

Thank you for this comment. Despite our sample size being comparable with other studies in the field (Lederman et al, 1990; Bara, 2014; Heller, 1996), we agree that the sample size of early blind children participated in two experiments is a limitation to the generalization of the findings. Hence, we added a limitation paragraph at the discussion to point this issue:

Page 25, line 524: A limitation to the generalization of the findings reported here, however, is the small sample size of early blind children included in the two experiments. Indeed, gathering samples of sufficient size is a common challenge for researchers working in the field of visual impairment [45, 46]. The population of people with visual impairments is characterized by wide interpersonal variability due to the type of visual pathology and age of diagnosis. There is a small number of adults and children who are completely blind since birth or since the first few years of life, and some have pathologies entailing additional handicaps. Thus, instead of trying to gather large samples of subjects at risk for having a large interpersonal variability, we found it more relevant to have two small samples with a larger exclusion criterion. This enables us to have corresponding inter-group profiles with regard to the age of diagnosis of complete blindness (early blind), no additional handicaps and educational level. Without intending to statistically compare results from the two experiments, the profile match between the two groups provides an overview of the effects of two techniques of illustration on early blind and sighted childrens' identification of the same objects. Although our simple size is comparable with other studies in the field assessing recognition of tactile content by blind children and adults [2, 23, 47], findings need to be taken into account carefully.

Reviewer #1/Minor issues : Please avoid using terms like "the blind" as they tend to define a person by their disability. Use instead terms like "blind people" or "people who are blind" (http://interactions.acm.org/archive/view/november-december-2015/writing-about-accessibility) :

Line 37: "The blind" changed for "blind people"

Line 156: "the sighted and the blind simulations" changed for "the sighted and blind adults' simulations"

Line 240: the blind changed for "blind people"

83: Close parenthesis:

Line 83, Corrected

108: This idea is using 3D:

Line 108: Corrected, "the idea is to use"

Reviewer #2: I suggest to authors to add recent studies on sensory integration in typically and visually impaired children (e.g. Cappagli et al., 2019, Gori et al. 2010).

Answer:

Thank you very much for this suggestion. Indeed, our study is in dialogue with the proposed references, which show the importance of multisensory calibration in a context of blindness. We have included these studies in the introduction:

Page 6, line 129 : Furthermore, studies on sensory integration [37, 38] have shown that lack of vision can alter the intermodal calibration necessary for spatial discrimination in blind children [for a review see 39]. Thus, compensation via other intact senses associating for example auditory and motor information can help blind children to process spatial information [38]. Gappagli and colleagues have tested a technological device (ABBI - Audio Bracelet for Blind Interaction), which emits a sound when a movement occurs, with 3-7 year-old children with congenital blindness or low vision. After 3 months of training, authors found that the spatial performance of congenitally blind but not low vision children is improved. Results indicated that in the absence of sight, the association of sensorimotor data with other intact senses is of great importance.

37. Gori M, Sandini G, Martinoli C, Burr D. Impairment of auditory spatial localization in congenitally blind human subjects. Brain. 2014;137(1):288-93. doi: doi.org/10.1093/brain/awt311.

38. Cappagli G, Finocchietti S, Baud-Bovy G, Cocchi E, Gori M. Multisensory rehabilitation training improves spatial perception in totally but not partially visually deprived children. Frontiers in Integrative Neuroscience. 2017;11. doi: doi.org/10.3389/fnint.2017.00029.
39. Valente D, Bara F, Afonso Jaco A, Baltenneck N, Gentaz E. La perception tactile des propriétés spatiales des objets chez les personnes aveugles. Enfance. in press.
